# Relationship of Creatinine and Nutrition with Arsenic Metabolism

**DOI:** 10.1289/ehp.1104807

**Published:** 2012-04-01

**Authors:** Mary V. Gamble, Megan N. Hall

**Affiliations:** Department of Environmental Health Sciences, Mailman School of Public Health, Columbia University, New York, New York, E-mail: Mvg7@columbia.edu; Department of Epidemiology, Mailman School of Public Health, Columbia University, New York, New York, E-mail: Mh2825@columbia.edu

Basu et al. (2011) reported the associations of both dietary and blood nutrient measures, as well as urinary creatinine (uCr), with arsenic (As) methylation capacity, as assessed by the proportions of urinary inorganic, monomethyl, and dimethyl As metabolites. One finding was that uCr was the strongest predictor of As methylation; participants with higher uCr concentrations had a higher percentage of total urinary As as dimethylarsinic acid (DMA) compared to those with lower uCr. This is consistent with what we have previously reported in Bangladeshi adults and children (Gamble et al. 2005; Ahsan et al. 2007; Hall et al. 2009), and is an interesting and potentially very important observation. Approximately 40% of *S*-adenosylmethionine (SAM)-derived methyl groups are devoted to the biosynthesis of creatine, the precursor of creatinine (Brosnan et al. 2011; Mudd and Poole 1975). At high levels of As exposure (500–1,000 µg/L), based on one-carbon kinetics (Schalinske and Steele 1989), we estimated that methylation of 80% of a daily dose of inorganic As (InAs) to DMA would require approximately 50 µmol SAM, thus consuming approximately 2–4% of the SAM normally turning over in a well-nourished adult per day. Low dietary creatine intake associated with low-protein or vegetarian diets places an increased demand for SAM for creatine biosynthesis (Brosnan 2011). This could potentially reduce the availability of SAM for As methylation, providing a plausible mechanism underlying this highly reproducible observation. This assumes that uCr reflects, to some extent, dietary creatine intake, as we have observed (Gamble M, unpublished data). Conversely, dietary creatine intake and/or creatine supplementation down-regulates endogenous creatine biosynthesis, potentially sparing SAM for methylation of other substrates such as As. We are currently testing this hypothesis in a randomized controlled trial of creatine supplementation. In addition, as Basu et al. (2011) noted, and as we have previously reported (Gamble and Liu 2005), one implication of the observed association between uCr and As methylation capacity is that urinary As should not be expressed per gram creatinine to correct for urine concentration. Rather, uCr should be included as a covariate in regression models.

One concerning aspect of the study by Basu et al. (2011) is the handling of blood samples used for nutrient measurements. As noted by Basu et al. and in a previous publication on these same participants (Chung et al. 2006), the blood samples were stored in an ice chest in the field for up to 24 hr before processing. This 24-hr delay can be problematic for some nutrients, especially folate, which is extremely sensitive to oxidative degradation (Drammeh et al. 2008). Basu et al. (2011) reported that in univariate analyses, they observed higher urinary percentages of InAs in individuals with higher serum folate concentrations. This finding is contrary to our previous findings that folate facilitates As methylation (Gamble et al. 2005, 2006, 2007; Hall et al. 2007, 2009). This discrepancy might be explained by differences in sample processing.

Basu et al. (2011) also reported associations between dietary intake of several nutrients (assessed using a modified 24-hr recall) and As methylation capacity. One of the most critical and widely discussed issues in nutritional epidemiology is the method used to adjust for total energy intake (TEI) (Willett et al. 1997). The main reasons to adjust for TEI are to *a*) adjust for potential confounding by TEI, *b*) remove extraneous variation in nutrient intakes that is due only to their correlation with TEI, and *c*) simulate a dietary intervention. What is often most relevant is diet composition, or nutrient intake in relation to TEI (Willett et al. 1997). Several methods are available to adjust for TEI, and the best approach can vary depending on the nutrient and question of interest. Basu et al. (2011) adjusted for TEI by dividing each nutrient intake by TEI (nutrient density method). While this approach is appealing because of its simplicity, in reality it can create a complex variable (Willett and Stampfer 1998). For example, when TEI is related to the outcome of interest, the use of nutrient densities can actually induce confounding in the opposite direction. Although we cannot determine from Basu et al.’s article whether TEI measured by the 24-hr recall was associated with As methylation, in theory, an association seems plausible. Also, because their statistical analysis tested for associations between multiple nutrients and urinary As metabolites, it is best to acknowledge that some of the statistically significant associations might be due to chance alone.

## References

[r1] Ahsan H, Chen Y, Kibriya MG, Slavkovich V, Parvez F, Jasmine F (2007). Arsenic metabolism, genetic susceptibility, and risk of premalignant skin lesions in Bangladesh.. Cancer Epidemiol Biomarkers Prev.

[r2] Basu A, Mitra S, Chung J, Guha Mazumder DN, Ghose N, Kalman DA (2011). Creatinine, diet, micronutrients, and arsenic methylation in West Bengal, India.. Environ Health Perspect.

[r3] Brosnan JT, da Silva RP, Brosnan ME (2011). The metabolic burden of creatine synthesis.. Amino Acids.

[r4] Chung JS, Haque R, Guha Mazumder DN, Moore LE, Ghosh N, Samanta S (2006). Blood concentrations of methionine, selenium, beta-carotene, and other micronutrients in a case-control study of arsenic-induced skin lesions in West Bengal, India.. Environ Res.

[r5] Drammeh BS, Schleicher RL, Pfeiffer CM, Jain RB, Zhang M, Nguyen PH (2008). Effects of delayed sample processing and freezing on serum concentrations of selected nutritional indicators.. Clin Chem.

[r6] Gamble MV, Liu X (2005). Urinary creatinine and arsenic metabolism. Environ Health Perspect.

[r7] Gamble MV, Liu X, Ahsan H, Pilsner R, Ilievski V, Slavkovich V (2005). Folate, homocysteine, and arsenic metabolism in arsenic-exposed individuals in Bangladesh.. Environ Health Perspect.

[r8] Gamble MV, Liu X, Ahsan H, Pilsner JR, Ilievski V, Slavkovich V (2006). Folate and arsenic metabolism: a double-blind, placebo-controlled folic acid-supplementation trial in Bangladesh.. Am J Clin Nutr.

[r9] Gamble MV, Liu X, Slavkovich V, Pilsner JR, Ilievski V, Factor-Litvak P (2007). Folic acid supplementation lowers blood arsenic.. Am J Clin Nutr.

[r10] Hall M, Gamble M, Slavkovich V, Liu X, Levy D, Cheng Z (2007). Determinants of arsenic metabolism: blood arsenic metabolites, plasma folate, cobalamin, and homocysteine concentrations in maternal–newborn pairs.. Environ Health Perspect.

[r11] Hall MN, Liu X, Slavkovich V, Ilievski V, Pilsner JR, Alam S (2009). Folate, cobalamin, cysteine, homocysteine, and arsenic metabolism among children in Bangladesh.. Environ Health Perspect.

[r12] Mudd SH, Poole JR (1975). Labile methyl balances for normal humans on various dietary regimens.. Metabolism.

[r13] Schalinske KL, Steele RD (1989). Quantitation of carbon flow through the hepatic folate-dependent one-carbon pool in rats.. Arch Biochem Biophys.

[r14] Willett WC, Howe GR, Kushi LH (1997). Adjustment for total energy intake in epidemiologic studies.. Am J Clin Nutr.

[r15] Willett W, Stampfer M (1998). Implications of total energy intake for epidemiologic analysis.

